# Quantitative translational modeling to facilitate preclinical to clinical efficacy & toxicity translation in oncology

**DOI:** 10.4155/fsoa-2017-0152

**Published:** 2018-04-23

**Authors:** Andy ZX Zhu

**Affiliations:** 1 Department of Drug Metabolism & Pharmacokinetics, Takeda Pharmaceuticals International Co., 35 Lansdowne Street, Cambridge, MA 02139, USA

**Keywords:** cancer growth modeling, drug combination, GRI, myelosuppression, pharmacokinetics, PK/PD, QSP, toxicity, translational, xenograft

## Abstract

Significant scientific advances in biomedical research have expanded our knowledge of the molecular basis of carcinogenesis, mechanisms of cancer growth, and the importance of the cancer immunity cycle. However, despite scientific advances in the understanding of cancer biology, the success rate of oncology drug development remains the lowest among all therapeutic areas. In this review, some of the key translational drug development objectives in oncology will be outlined. The literature evidence of how mathematical modeling could be used to build a unifying framework to answer these questions will be summarized with recommendations on the strategies for building such a mathematical framework to facilitate the prediction of clinical efficacy and toxicity of investigational antineoplastic agents. Together, the literature evidence suggests that a rigorous and unifying preclinical to clinical translational framework based on mathematical models is extremely valuable for making go/no-go decisions in preclinical development, and for planning early clinical studies.

## Lay abstract

Significant scientific advances in biomedical research have expanded our knowledge of the molecular basis of carcinogenesis, mechanisms of cancer growth and the importance of the cancer immunity cycle. However, despite that in many cases drug treatment can eradicate tumors in animals, treating human tumors remains very difficult. This article describes a mathematical modeling framework to facilitate the prediction of clinical efficacy and toxicity of investigational antineoplastic agents.

Over the last 15 years, scientific advances in biomedical research have expanded our knowledge of the molecular basis of carcinogenesis, mechanisms of cancer growth and the importance of the cancer immunity cycle [1–3]. As a result of those advances, a number of transformative anticancer therapies have been developed, which have brought meaningful declines of mortality and morbidity to patients with this devastating disease. However, despite the scientific advances in the understanding of cancer biology, the success rate of oncology drug development remains the lowest among all therapeutic areas [4]. A recent analysis of 7455 clinical drug development programs between 2006 and 2015 showed that the likelihood of regulatory approval for a Phase I program is the lowest in oncology drug development [4]. Only 5–7% of Phase I clinical oncology programs and <50% of Phase III oncology programs are ultimately approved [5]. A major reason behind this low success rate is the lack of a unifying framework to predict clinical efficacy and toxicity profiles from animal and *in vitro* experiments. A rigorous unifying preclinical to clinical translational framework could facilitate oncology clinical development by better identifying translational strategies, patient selection criteria and appropriate biomarkers to measure [6]. Robust translational research will help with making informed decision early in drug development and improve success rate of late stage programs.

In this review, evidence will be presented to demonstrate that mathematical modeling can be used to build a rigorous and unifying preclinical to clinical translational framework to understand the anticipated exposure–response relationship in humans while considering the tolerability profile. This information can be used to understand the benefit–risk profile of the investigational drug in different patient populations and maximize the drug's potential in clinical development.

Historically, the development of antineoplastic agents is largely an empirical trial-and-error process. This was particularly true for antineoplastic drugs with cytotoxic mechanisms of action since these drugs are often dosed at the maximum tolerated dose (MTD) in humans and this information was generally obtained experimentally in dose escalation studies.

However, as a result of recent advancement in molecularly targeted therapy, immuno-oncology drugs, cancer vaccines, cell-based therapies, and endless possibilities of drug combinations, obtaining an optimal toxicity–efficacy balance is an increasingly complex task since not all drugs are dosed at MTD. Consequently, the standard empirical approaches used in the past to optimize drug dosing and scheduling in patients are now of limited utility. A more rational dose selection process using mathematical modeling, which is built on a clear understanding of the target biology, to determine the required degree of target engagement would be extremely valuable and could potentially save a lot of time since patient recruitment is challenging for Phase I oncology trials. This mathematical framework can also be easily updated with clinical data and subsequently used to refine drug dosing and scheduling as well as guide go/no-go decisions and trial designs [7].

The scientific and regulatory fields have long recognized the utility of mathematical modeling framework. As early as 2006, the US FDA critical path opportunities report advocated the use of modeling and simulation for decision making in drug development [8]. More recently, the FDA reinforced this idea in their FDA voice blog, which states: “Modeling and simulation play a critical role in organizing diverse datasets and exploring alternate study designs. This enables safe and effective new therapeutics to advance more efficiently through the different stages of clinical trials” [9].

In this review, some of the key translational drug development objectives in oncology will be outlined. Additionally, the literature evidence of how mathematical modeling could be used to build a unifying framework to answer these questions will be summarized. Some recommendations for strategies to build a mathematical modeling framework that facilitates the prediction of clinical efficacy and toxicity of investigational antineoplastic agents will also be discussed.

## Key translational objectives of a preclinical oncology drug development program

Generally, there are two key objectives for a standard preclinical oncology drug discovery program. Firstly, the preclinical program needs to provide safety data to support an appropriate starting dose for Phase I clinical programs. This is traditionally achieved using *in vivo* animal toxicology studies. Secondly, the preclinical program needs to provide scientific support for the rationale and biological plausibility of the investigational drug to warrant a clinical study.

A major challenge to achieve these preclinical objectives is to determine the cross-species differences and the relevance of the preclinical efficacy and toxicity data. Mathematical modeling and simulation can be used to account for the species differences and collect all available data to make quantitative predictions about the therapeutic index of the investigational agent in humans. This allows decisions to be made accordingly on whether to advance this drug further into clinical development. The same mathematical model can also be used to determine the appropriate starting dose, the projected human efficacious dose and the appropriate dosing schedule to be evaluated in Phase I trials. This greatly maximizes safety and minimizes toxicity during Phase I studies and align realistic expectations of the drug efficacy. The detailed mathematical approaches to build a translational modeling framework are described in the subsequent sections.

## Translating preclinical antitumor activities to clinical efficacy

Due to ethical and practical consideration associated with human clinical studies, animal models have been used extensively in oncology research to evaluate the activities of antineoplastic agents. In contrast to other therapeutic areas such as psychiatry, preclinical oncology research extensively utilizes human tissues [10]. The use of human tissues minimizes the need of accounting for species-specific biology in efficacy translation. In fact, the most commonly used preclinical model in oncology is the mice xenograft model, which comprises subcutaneous implantation of a human cell line/tumor into an immune-compromised host mice [11,12]. The xenograft model represents extreme simplification of human cancer, as it does not account for the complexities of tumor metastasis, host immunity, tumor heterogeneity, and the development of treatment resistance that is routinely observed in cancer patients. [13,14]. Nevertheless, the drug exposure–response relationship derived from these models is still useful for understanding the degree of antitumor activity associated with the investigational drug, and allows *in vivo* interpretation of tumor growth inhibition data to inform early clinical development [15]. In a typical xenograft experiment, the xenograft tumor volume is measured over time after drug treatments using calipers. The drug-treated tumor volume profile is then compared with that of the vehicle treatment to obtain a quantitative metric of antitumor activity associated with the investigational drug. This information can be used in mathematical models to define efficacy and predict clinical antitumor response.

Due to the variability in the growth rate of human tumors in mice and the small sample sizes of preclinical experiments, rigorous mathematical and statistical analyses are critical in preclinical drug development. There are a number of mathematical approaches to translate the preclinically observed antitumor activity into clinical efficacy. These approaches can generally be categorized into static algebraic approaches and dynamic, differential equation-based approaches. The most commonly used approaches for efficacy translation are discussed in the next section.

### Static algebraic approaches of characterizing antitumor activity in preclinical models

**Figure 1.**
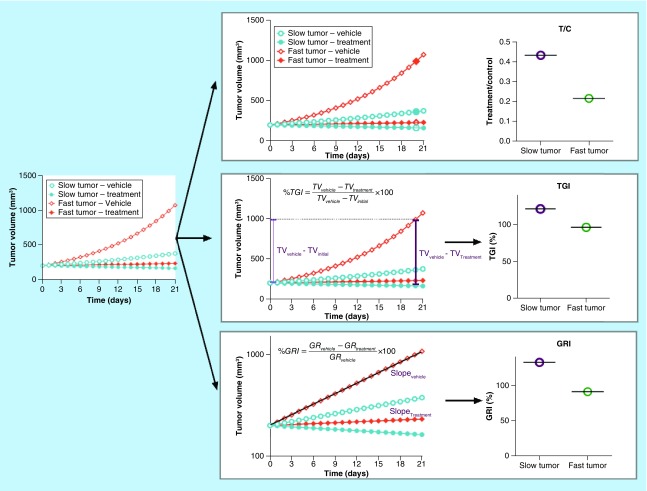
**Commonly used antitumor efficacy metrics.** Top panel: T/C ratio. Middle panel: TGI. Bottom panel: GRI. T/C overestimates the antitumor activity of the fast-growing tumors compared with the slow-growing tumors, which significantly limits its value for predicting clinical efficacy. TGI is generally less dependent on the tumor growth rate than T/C. GRI, which is calculated by fitting all available tumor volume data first to an exponential growth function, is the least dependent on the intrinsic growth rate of the tumor. GRI: Growth rate inhibition; T/C: Tumor volume over control volume; TGI: Tumor growth inhibition.

There are three static algebraic approaches of antitumor activity that are commonly used in xenograft experiments: tumor volume over control volume (T/C ratio); tumor growth inhibition (TGI); and growth rate inhibition (GRI). Their calculations are summarized in Figure 1. Although there are advantages and disadvantages associated with each algebraic descriptor, GRI is the least dependent on the tumor growth rate and the design of xenograft experiments. Therefore, GRI should be considered as the first choice for translational work. The subsequent paragraphs will introduce each of the three approaches and summarize the advantages of GRI.

T/C ratio is an easy-to-calculate metric of antitumor activity and is often used preclinically to characterize antitumor activity [16–18]. T/C ratio is calculated by dividing the tumor volume of the drug-treated group by the tumor volume of the vehicle-treated group at a predefined time (typically 3 weeks after starting the treatment, illustrated by Figure 1). Although it is easy to calculate, the T/C ratio has some significant limitations since it is heavily influenced by the natural growth rate of the xenograft tumor and the time points at which the T/C ratio is calculated. This limitation is illustrated by the top panel of Figure 1. When treatments of both the slow and fast-growing tumors result in tumor stasis, the T/C ratio generally overestimates the antitumor activity of the fast-growing tumors in comparison to the slow-growing tumors. Due to this undesirable property, the T/C ratio has very limited value for predicting human antitumor efficacy from preclinical data.

TGI is another commonly used static metric to measure antitumor activities (Figure 1, middle panel) [12,19,20]. It is calculated by dividing the tumor volume difference between the vehicle group and the drug-treated group by the tumor volume difference between the vehicle group and the tumor's initial volume (Equation 1). TGI is generally less dependent on the tumor growth rate. Additionally, the TGI obtained from mice subcutaneous tumor models has been show to correlate with human clinical overall response rate for a spectrum of antineoplastic agents [12]. However, it still has a few limitations compared with the GRI.(Equation 1)
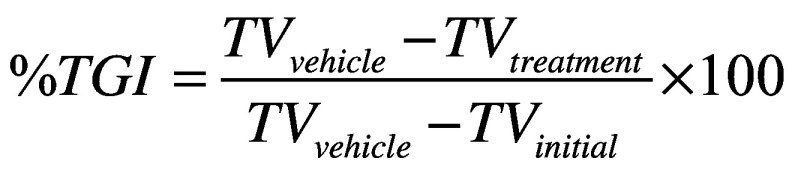



In the last few years, GRI, a novel rate-based T/C metric, has become more popular for characterizing the preclinical antitumor activity [21]. GRI is calculated by fitting all available tumor volume data first to an exponential growth function. The resulting growth rates under the treatment and control conditions are then used to calculate the percentage of GRI (Equation 2).(Equation 2)
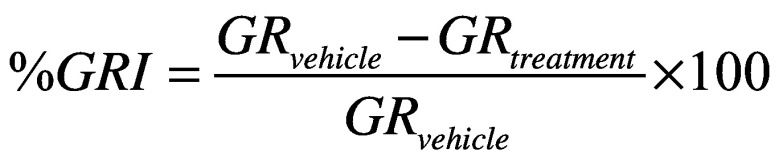



**Figure 2.**
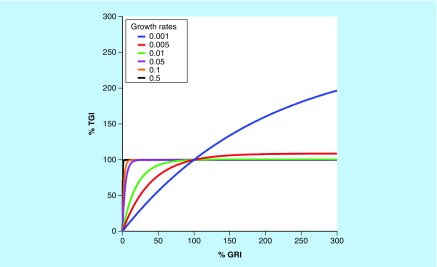
**Correlation between tumor growth inhibition and growth rate inhibition for xenograft tumors of different growth rates.** Tumor growth inhibition and growth rate inhibition show good correlations for slow-growing xenograft tumors. For fast-growing xenograft tumors, growth rate inhibition has a much more dynamic range compared with the tumor growth inhibition, which saturates at around 100%. GRI: Growth rate inhibition; TGI: Tumor growth inhibition.

There are a few advantages of using GRI to characterize antitumor activities. Firstly, since all available tumor volume data are used to fit the exponential function, GRI is more efficient than the T/C ratio and TGI; it requires fewer animals to achieve the same statistical power – as little as six animals per group would have sufficient statistical power for translation [21]. Secondly, theoretical simulations have suggested that GRI can tolerate shorter study durations compared with the T/C ratio and TGI [21]. Thirdly, compared with T/C or TGI, GRI is less influenced by the intrinsic growth rate of the xenograft tumor. Therefore, it is ideally suited to compare the drug efficacies across different xenograft models. In particular, GRI has a much more dynamic range compared with TGI in fast-growing tumors. As illustrated by Figure 2, for xenograft tumors which are slower growing (i.e., smaller growth rate), GRI and TGI show a good correlation. However, for xenograft tumors with faster growth rates, GRI has a much more dynamic range compared with TGI, which saturates at around 100% in fast-growing tumors.

### Dynamic differential equation-based approaches of characterizing antitumor activity in preclinical models

Although static algebraic approaches are very useful for summarizing the antitumor activity, they often cannot be used to describe complex exposure–response relationships such as sigmoidal or Michaelis–Menten type of dose–response curves. Furthermore, there is often a delay between drug administration and tumor shrinkage (i.e., it takes a while for the drug to kill the tumor cells) which cannot be easily accounted for by static approaches. To overcome these limitations, a number of differential equation-based dynamic approaches have been developed to describe the tumor growth and exposure–response relationship of antineoplastic agents. The common approaches are summarized in Table 1. In general, the dynamic approaches can be categorized into two groups: phase-specific models and phase nonspecific models. The phase-specific models assume that cancer cells are only susceptible to the antineoplastic agents at a specific stage of the cell cycle (for example, antimitotic agents only work on cancer cells that are dividing). These models typically include a series of transit compartments (Table 1) and usually provide a good fitting of observed data [22–25]. In contrast, the phase nonspecific models do not use transit compartments and generally do not fit the observed delay between drug administration and tumor shrinkage well [12,26–28]. Another key aspect of dynamic models is the form of the kill function, which can be either linear, Michaelis–Menten or sigmoidal. The mathematical form of this function dictates whether the model can be used to understand the impact of drug schedule (Table 1). A detailed tutorial has been published to discuss the proper use of dynamic models [29].

**Table 1.** **Commonly used pharmacokinetic/pharmacodynamics models to describe tumor growth kinetics.**

**Study (year)**	**Tumor growth term**	**Tumor kill term**	**Transit compartments**	**Comments**	**Ref.**
**Phase nonspecific models**					

Kogame *et al*. (2013)			0	This model was developed for a potent, selective hedgehog signaling pathway inhibitor. It was used to identify the degree of PD inhibition required to inhibit tumor growth.	[26]

Yamazaki (2008)			0	This model was developed to understand the PK/PD relationship of a small molecule cMet inhibitor. The model structure closely resembles an indirect response model.	[28]

Wong *et al*. (2009)			0	This model was developed to understand the PK/PD relationship of a B-Raf inhibitor. No transit compartments were used.	[30]

Salphati (2010)			0	This model was developed understand the PK/PD relationship of a PI3K inhibitor. This model used a hill coefficient on the effect term which is very unique.	[27]

**Phase-specific models**					

Lobo *et al*. (2002)			3	One of the first PK/PD models utilizing transit compartment to describe the antitumor. It was initially developed for methotrexate.	[22]

Simeoni *et al*. (2004)			3	This is one of the most commonly used model structure for modeling preclinical antitumor activity. It was initially validated against a few cytotoxic agents, but the structure has been subsequently applied to many other types of compounds.	[23]

Bueno *et al*. (2008)		Effect = biomarker	2	It was developed for LY2157299, a new type 1 receptor TGF-β antagonist. Tumor growth inhibition was linked to PD biomarker level.	[24]

Jumbe *et al*. (2010)			2	This model was developed for trastuzumab-DM1 (an ADC) in order to determine the optimal dose and dosing schedule for antitumor activity.	[25]

ADC: Antibody–drug conjugate; PK/PD: Pharmacokinetic/pharmacodynamics.

### Using xenograft antitumor activity to predict antitumor activity in humans

**Figure 3.**
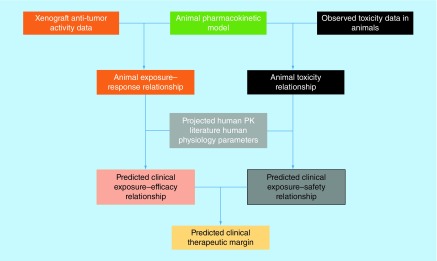
**Common translational modeling approach for predicting the efficacy potential of an investigational antineoplastic agent.** Firstly, a pharmacokinetic mathematical model needs to be constructed as a foundation, based on pharmacokinetic measurements in mice. Secondly, xenograft efficacy studies are used to establish an exposure–response relationship. Thirdly, the xenograft exposure–response relationship is translated into humans based on human data. Lastly, a translational exposure-tolerability model based on animal and human toxicity data is used to predict whether the drug would have a meaningful tumor regression in humans at a tolerable dose. PK: Pharmacokinetic.

Whether the xenograft transplantation of subcutaneous tumor into immune-deficient mice provides predictive values for discerning clinically efficacious antineoplastic agents has long been debated. Mathematical models of preclinical antitumor data can play an important role in understanding the clinical potential of an investigational antineoplastic agent. The translational modeling of preclinical antitumor activity data usually is a four-step process (Figure 3). Firstly, a robust pharmacokinetic (PK) mathematical model, describing the concentration dynamic of the drug, needs to be constructed based on PK measurements in mice (ideally in the same type of xenograft mice as the efficacy studies). Secondly, using the established mice PK model as a foundation, an exposure–response relationship can be established using xenograft efficacy studies to understand the effects of different doses/schedules on the time course of tumor growth. The exposure–response relationship can be either static (as expressed by a correlation between drug exposure and the resulting GRI on a graph) or dynamic (as expressed using a set of differential equations to account for delayed drug effects and changes in growth rate over time) in nature [23]. A key consideration at this stage is selecting the appropriate xenograft model for clinical translations. This is particularly important for molecularly targeted therapy (such as HER2 or EGFR inhibitors), as it is essential to ensure that the xenograft model contains the pathway activation phenotype relevant to the drug's mechanism of action. The third step in the translation process is to translate the xenograft exposure–response relationship into humans by substituting the PK portion of the mice model with predicted or observed human PK parameters, depending on whether observed human data are available. A large body of literature exists for human PK predictions of small and large molecules and has been reviewed extensively elsewhere [31–35]. In this step, a key consideration is to incorporate interspecies differences in plasma protein binding to ensure that the translation is based on the free fraction of the drug. After completing this step, a mathematical model can be used to predict the human exposure–response relationship and simulate the clinical dose/schedule effects on tumor growth. The last step is to use a translational exposure-tolerability model to predict the maximum tolerable dose/exposure in humans and evaluate whether the investigational drug would have a meaningful tumor regression in humans at a tolerable dose. This step is also very important since one of the major reasons for the lack of translatability is that mice often can tolerate much higher drug exposure compared with humans. Therefore, even if an antineoplastic agent shows good efficacy in the preclinical model, the efficacious exposure may not be safely achieved in humans. In fact, a poor correlation is observed between the antitumor activity at mice MTD and activity in the clinic, suggesting that proper anticipation of human tolerability is essential for this kind of translational work [12].

**Figure 4.**
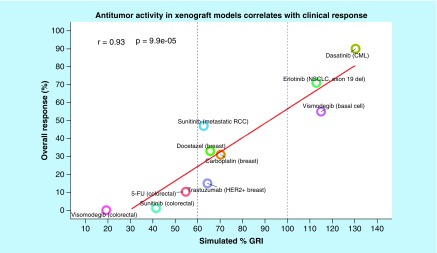
**Antitumor activity in xenograft correlates with clinical response.** The data were collected from the literature and percentage growth rate inhibition was calculated using digitalized data [12]. Antitumor activity in xenograft correlates with clinical response for a spectrum of molecularly targeted and cytotoxic agents at clinical maximum tolerable exposure. GRI: Growth rate inhibition; RCC: Renal cell carcinoma.

More recent research shows that preclinically predicted antitumor activity at human tolerable exposures correlated strongly with clinical response [12]. Figure 4 summarizes the correlation between the preclinical predicted antitumor activity (percentage GRI) and overall clinical response rate for a spectrum of molecularly targeted and cytotoxic agents at clinical maximum tolerable exposure. The data were collected from the literature and percentage GRI was calculated using digitalized data [12]. This analysis suggests that when proper mathematical models are used to account for human tolerable exposures, preclinical antitumor activity is highly predictive of the overall response rate in the clinic. This strongly suggests that xenograft transplantation of subcutaneous tumor into immune-deficient mice can be used to discern the clinical potential of novel antineoplastic agents and highlights the importance of using proper mathematical models for preclinical to clinical translation.

Using the modeling framework summarized in Figure 3, a number of successful preclinical–clinical translation examples have been demonstrated [20,26,36–44]. In the case of cytotoxic agents, Jumbe *et al*. described a cell-cycle phase nonspecific tumor cell kill model which captured the features of tumor growth in trastuzumab-DM1-treated animals under a number of single-dose, multiple-dose, and time–dose–fractionation conditions. This model suggested that the antitumor activity was schedule-independent, and the tumor response was determined by the ratio of drug exposure to a critical tumor stasis concentration [25]. This modeling framework was also used to translate the molecularly targeted agents. For example, Tate *et al*. described a PK–pharmacodynamic-efficacy (PK–PD-E) model for the translation of an investigational cyclin-dependent kinase 4/6 inhibitor. This model dynamically linked drug concentrations to pharmacodynamic effects such as decreased phosphorylation of retinoblastoma protein, cell-cycle arrest, and tumor growth inhibition. The model was used to support the clinical chronic dosing regimen for durable cell-cycle inhibition [37].

Although the exposure–response relationship usually translates well for cytotoxic agents and molecularly targeted agents, its utility is more limited in the immuno-oncology field since there are some fundamental differences between human and mice immune systems. In this case, quantitative system pharmacology (QSP) models, which link PK with mechanistic models of biological pathway modulation, become extremely useful. Since these models are built on the understanding of the biological pathways and the model parameters are often generated experimentally rather than fitted empirically, they can be viewed as quantification of the underlying biological processes. QSP models enable the separation of biological and drug specific parameters, and thus have an enhanced interspecies translational ability. This property is ideally suited for translational modeling of immuno-oncology agents since one could re-parameterize the model using human-specific biological parameters while keeping the drug-specific parameters the same between mice and humans since these are properties of the drug.

Several publications investigated the translatability of immuno-oncology agents using this QSP framework. For example, Lindauer *et al*. built a quantitative system pharmacology model to identify the lowest effective dose for evaluation in clinical dose-finding studies for pembrolizumab, a programmed death 1 checkpoint inhibitor. This model linked a compartmental PK model to a published physiologically based tissue compartment and used receptor occupancy as the driver of observed tumor growth inhibition [45]. A similar QSP-based model was also used to predict the safe starting dose and clinical efficacious dose for P-cadherin LP-DART, a bispecific T-cell engager [40]. Overall, although a relatively new concept, the QSP modeling field is growing rapidly and more case examples will become available in the coming years to really explore its full potential.

### Methods for predicting clinical efficacy of drug combinations

Another very important aspect of modern oncology research is to understand drug combinations. Mathematical modeling of preclinical data can also provide guidance on the predicted clinical benefit of combination therapies under different dose and scheduling combinations. This is particularly important since combinations can be extremely complex when all permutations of doses and schedules are considered and testing all combinations in the clinic is simply not possible.

The antitumor activities of combination therapies can be modeled by either static or dynamic approaches. Static translations of antitumor activities are typically done via isobolograms of *in vivo* exposures at various levels of xenograft antitumor activities [46]. This approach is visual and could provide a graphical representation of the exposure–response relationship for drug combinations to guide dose selection and escalation in the clinic. The disadvantage of this isobolograms-based static approach is that it does not consider the time dynamics of drug concentrations and the dynamics of drug effects. A dynamic combination modeling framework has been published to better understand the sequential effect and prioritization of dose pairs (e.g., low-dose drug A + high-dose drug B vs high-dose drug A + low-dose drug B) [47]. This dynamic semimechanistic model allows the optimization of combination dosing schedules and determines which combination schedule would give the highest degree of synergy. The application of combination modeling to guide clinical dosing schedules has been described for combinations of MAPK and PI3K pathway inhibitors [48].

## Modeling translation of drug-induced toxicity

In addition to translating antitumor efficacies, mathematical modeling could also facilitate the quantitative prediction of exposure–safety relationship in order to gain a scientific understanding of the tolerability profile. Although antineoplastic agents are associated with many different types of toxicities/adverse events, some of the most common toxicities can be readily described by mathematical models. Properly verified mathematical models can be used to ask the what-if questions related to drug safety. For example, mathematical modeling can be used to assess the impact of intrinsic or extrinsic factors, such as organ impairment, gender, race and drug–drug interaction, on the safety profile. Some of the most mature translational mathematical models are in the areas of myelosuppression, gastrointestinal (GI) toxicity, and cardiac safety. The current state of scientific knowledge is discussed below.

### Neutropenia & general myelosuppression models

Antineoplastic agents often have the greatest impact on the growth and survival of rapidly proliferating cells. One of the most notable rapidly proliferating cell types in the body is the hematopoietic stem cell (HSC). In a person's life time, HSCs, which account for just 0.01–0.2% of the total bone marrow mononuclear cells, produce blood cells weighing ten-times more than the bodyweight of that person [49,50]. Due to their high proliferative potential, cells derived from HSC are extremely susceptible to the cytotoxic effects of antineoplastic agents.

As all blood cells, neutrophils are produced by HSC in the bone marrow. Neutrophils are short lived in the blood circulation [51] and their level is dependent on a constant state of production [52]. Thus, neutrophils’ homeostasis is particularly susceptible to antineoplastic agents [52]. Neutropenia (low neutrophil counts) and febrile neutropenia increase the risk of hospitalization and complications as a result of an increased susceptibility to infections. Therefore, it has become a primary concern in oncology drug development. A general myelosuppression model, which can be applied to neutropenia, was one of the first mathematical models developed to characterize the time courses of adverse events and has been used to understand the exposure–safety relationships of a huge number of antineoplastic agents [53].

This model mimics myelopoiesis by including a concentration-related drug effect on a proliferating precursor cell compartment. Maturation of the precursor cells in the bone marrow is modeled as a series of transit compartment to explain the delayed effect on circulating neutrophil counts. A homeostatic feedback mechanism is included to stimulate the increase in proliferation of the precursor cells when circulating mature neutrophils counts are low. The attractiveness of this model for translation lies in its ability to separate drug-related versus physiology-related processes, which allows for predictions of untested scenarios in different species. Due to its parsimonious structure, it is frequently applied and has been shown to be highly reproducible and robust [53]. Due to its simplistic nature, this mechanism-based structure also allows for extensions, such as rescue treatments with recombinant granulocyte-colony stimulating factors.

In the context of preclinical to clinical translation of drug safety, this model separates the drug- and physiology-related processes. Since species-dependent parameters can be obtained from the literature, only drug-specific potency parameters are needed to predict the clinical myelosuppression profile from preclinical data. It has been shown that drug-specific potency parameters can be scaled from animal to humans after correcting for species differences in protein binding and *in vitro* sensitivity [54]. Using this kind of translational exposure safety model, the tolerability profile in Phase I studies can be forecasted and updated to increase precision once neutrophil data in humans become available. This strategy allows more rapid decision making on dosing schedules and dose escalation [55,56]. Furthermore, although neutropenia *per se* is not life threatening, it could result in reduced capability for fighting infections and the development of febrile neutropenia. A body of research has shown that the shape of neutrophil profile is related to the risk of developing febrile neutropenia [57]. A translational understanding of the risk could be invaluable in early clinical developments for identifying patients requiring rescue treatments and maximizing the therapeutic potential of the investigational agent [58,59].

### Gastrointestinal toxicity models

In addition to blood cells, enterocytes in the GI tract is another rapidly proliferating cell type in the body. Due to their high proliferative potential, enterocytes are also extremely susceptible to the cytotoxic effects of antineoplastic agents such as DNA-damaging agents, antimitotics, drugs which affect the protein homeostasis pathways and epigenetic regulators [60,61]. In fact, about 50–80% of the patients receiving chemotherapies develop GI-related adverse events [62,63]. The incidences and severity of GI-related adverse events could be potentially mitigated by changing the dosing schedule and employing dosing holidays in order to achieve efficacious exposures while maintaining tolerability [60,64]. The current dosing schedules for many antineoplastic agents are often identified empirically through head to head comparison in clinical trials. However, due to ethical, cost and time duration considerations, only a limited number of schedules could be tested in the clinic. Thus, a mechanistic understanding of GI toxicity using preclinical models and a quantitative translational framework could be extremely beneficial for the management of GI toxicity.

Although the molecular mechanisms regulating the homeostasis of intestinal epithelium are not as well understood as myelopoiesis, the fundamental process maintaining intestinal mucosa integrity has been described. Slowly dividing stem cells located near crypt bottoms produce rapidly dividing progenitor cells, which form mature absorptive cells, enterocytes and secretory cells after lineage commitment [65–67]. Mature epithelial cells then migrate toward the lumen side of the mucosa where they undergo apoptosis and shedding [66]. A number of system biology models have been constructed to investigate this process [68–72]. And more recently, the quantitative translational aspects of gastrointestinal toxicity are investigated using irinotecan [73]. Irinotecan is known to induce gastrointestinal-related adverse events in the clinic [74]. Shankaran *et al*. built a quantitative system pharmacology model to describe the key aspects of intestinal cell dynamics. This model was used to determine the toxicity of the compounds against intestinal crypts in rats and subsequently translated into quantitative predictions of enterocyte loss and recovery kinetics in humans [73]. The model predictions showed good correlation with clinical observed rate of irinotecan-induced gastrointestinal adverse events. The model was then used to simulate a range of clinical schedules to rank the schedules based on the extent of gastrointestinal toxicity [73]. This kind of translational work could be very beneficial for optimizing dosing schedule of cytotoxic agents. For other classes of antineoplastic agents such as immuno-oncology agents, animal models may not be relevant since the mechanism of GI toxicity is often not due to cytotoxicity in humans. For example, for nivolumab, pembrolizumab and ipilimumab, very limited GI toxicity was observed in animals but it is the dose-limiting toxicity in humans. Therefore, at this moment the GI toxicity prediction by mathematical models are more restricted to chemotherapeutic agents.

#### Other types of toxicity

In addition to myelosuppression and gastrointestinal toxicity, antineoplastic agents, particularly small molecule RTK inhibitors, also cause cardiovascular adverse events including QT prolongation. At a fundamental level, the heart is an electrical organ, and cardiac contractility is a complex interplay between many ion channels. This process can be recapitulated *in silico* through mathematical modeling with a good degree of certainty. In the pharmaceutical industry, significant investments have been made to develop mechanistic-based cardiac models to predict cardiac safety. The largest initiative is the Comprehensive *in vitro* Proarrhythmia Assay. Comprehensive *in vitro* Proarrhythmia Assay proposes to assess a drug's effect on multiple ion channels and integrate the effects in a computer model of the human cardiomyocyte to predict proarrhythmic risks [75]. The *in silico* reconstructions integrate drug effects on multiple human cardiac currents and the results are confirmed with human stem cell-derived cardiomyocytes. The modeling results could guide risk management in clinical trials, and in appropriate cases, potentially obviate the need for a dedicated QT study. Similar efforts are also under way to predict drug-induced liver injury, particularly in the bile acid transporter inhibition area [76,77].

## Conclusion & future perspective

We are in the midst of an evolving paradigm shift for oncology. The growing knowledge of basic molecular and cellular mechanisms underlying carcinogenesis and immuno-oncology as well as the availability of large amount of data will allow mathematical models to adequately describe the processes involved, and make quantitative predictions. The current mathematical modeling knowledge in oncology is largely built on the experience of developing cytotoxic and molecularly targeted agents. For immuno-oncology agents, syngeneic mice with intact immune systems are often used pre-clinically to determine the anti-tumor efficacy since the drug often targets the host immune system rather than the tumor itself. During the mathematical translation work, one would need to consider the species difference between mouse and human immune systems. This cross-species translation makes efficacy and toxicity projection much more difficult (Table 2). Making the cross-species translation even more difficult, the investigational drug often has different binding affinities in different species. Therefore, a key future direction for mathematical modeling in oncology is to build and validate translational immuno-oncology models. The oncology modeling field could do well by learning from the experience of vaccine and inflammation modeling community to solve these problems.

**Table 2.** **The key preclinical to clinical translational differences between cytotoxic antineoplastic agents and immuno-oncology agents.**

**Key translational differences**	**Cytotoxic agents**	**Immuno-oncology agents**
Preclinical model	Xenograft	Syngeneic

Target	Tumor	Host immune system

Benchmark for clinical efficacy	At least 60% GRI	–^†^

Efficacy response	Continuous and show dose response	Sometimes binary

Translation	Exposure-based translation	Biomarker-based translation/–^†^

FIH dose	1/6 of HNSTD	MABEL

^†^Unknown.

FIH: First in human; GRI: Growth rate inhibition; HNSTD: Highest nonseverely toxic dose; MABEL: Minimal anticipated biological effect levels.

Another future research direction is to understand the heterogeneity of antitumor responses. Due to practical limitations, only a small number of xenograft models could be studied in preclinical developments, and this limits our ability to understand how patients with heterogeneous tumor would respond to the investigational antineoplastic agent. Fortunately, significant advances have been made in the development of genomics technologies and high-throughput preclinical Phase II-like studies where hundreds of patient-derived xenograft tumors could be monitored for drug effects [78]. These data can be combined to determine the preclinical sensitivity differences between different types of molecular signatures [79,80]. Ultimately, this would allow better patient selection in the clinic and lead to more benefits for the patients. Patient-derived xenograft (PDX) models in the context of population pharmacokinetics/pharmacodynamics are a unique way of predicting population distribution of the responders and can be used as a Bayesian prior for clinical development. Furthermore, one of the major problems with current mice xenograft studies is the lack of genomic diversity. In the future, next-generation sequencing in high-throughput Phase II-like PDX studies will provide more information about how genomic diversity will impact drug pharmacodynamics and efficacy. If a predicted PD biomarker can be developed and incorporated into mathematical models, it could facilitate efficacy prediction in tumors of different genomic backgrounds.

Overall, despite many practical challenges, significant advances have been made to establish a mathematical modeling framework to facilitate the efficacy and toxicity translation in oncology. For cytotoxic agents and most of the molecularly targeted antineoplastic agents, mathematical modeling of the preclinical data can now predict the clinical efficacy and toxicity profile with good confidence as demonstrated by the examples discussed in this review. However, translation for immuno-oncology agents remains very difficult. The oncology translational modeling field should look to quantitative system pharmacology fields of inflammation and vaccines for scientific inspiration. The growing use of mathematical modeling in oncology translation will provide a unifying framework for evaluating the potential of an investigational oncology product. Lessons learned from the models could help determine the best clinical development strategy and the kinds of patients who would benefit the most from the new drug. Together, a model-based development paradigm will result in a rational and more efficient oncology drug development process.

**Executive summary**

- Over the last 15 years, scientific advances in biomedical research have expanded our knowledge of the molecular basis of carcinogenesis, mechanisms of cancer growth and the importance of the cancer immunity cycle. However, despite the scientific advances in the understanding of cancer biology, the success rate of oncology drug development remains the lowest among all therapeutic areas.
- Mathematical modeling can be used to translate preclinical knowledge into clinical predictions to refine drug dosing and scheduling as well as guide go/no-go decisions and trial designs.
- Preclinically, there are a number of mathematical approaches to translate the preclinically observed antitumor activity into clinical efficacy. These approaches can generally be categorized into static algebraic approaches and dynamic, differential equations-based approaches.
- Growth rate inhibition should be considered as the first choice for static algebraic translation of preclinical efficacy data.
- In the case of sigmoidal or Michaelis–Menten type of dose response curves or in a situation there is a delay between drug administration and tumor shrinkage. Dynamic differential equation-based dynamic approaches should be used to describe the exposure–response relationship.
- A four step process can be used to preclinical antitumor activity to clinical efficacy. Once the proper steps are taken, recent research shows that preclinically predicted antitumor activity at human tolerable exposures correlated strongly with clinical response.
- Mathematical modeling of preclinical data can also provide guidance on the predicted clinical benefit of combination therapies under different dose and scheduling combinations. Isobologram-based static approach and differential equation-based dynamic combination modeling framework have been published to better understand the sequential effect and prioritization of dose pairs.
- In addition to translating antitumor efficacies, mathematical modeling could also facilitate the quantitative prediction of exposure–safety relationship. Some of the most mature translational mathematical models are in the areas of myelosuppression, GI toxicity, and cardiac safety. The current state of scientific knowledge is discussed below.
- In summary, the growing use of mathematical modeling in oncology translation will provide a unifying framework for evaluating the potential of an investigational oncology product. Lessons learned from the models could help determine the best clinical development strategy and the kinds of patients who would benefit the most from the new drug. Together, a model-based development paradigm will result in a rational and more efficient oncology drug development process.

## References

[1] Hanahan D, Weinberg RA (2011). Hallmarks of cancer: the next generation.

[2] Hanahan D, Weinberg RA (2000). The hallmarks of cancer.

[3] Chen DS, Mellman I (2013). Oncology meets immunology: the cancer-immunity cycle.

[4] Mullard A (2016). Parsing clinical success rates.

[5] Ledford H (2011). Translational research: 4 ways to fix the clinical trial.

[6] Venkatakrishnan K, Friberg LE, Ouellet D (2015). Optimizing oncology therapeutics through quantitative translational and clinical pharmacology: challenges and opportunities.

[7] Sharma MR, Maitland ML, Ratain MJ (2012). Models of excellence: improving oncology drug development.

[8] US Department of Health and Human Services Food and Drug Administration (2006). Critical path opportunities report. http://www.fda.gov/ohrms/dockets/ac/07/briefing/2007-4329b_02_05_Critical%20Path%20Report%202006.pdf.

[9] Gottlieb S (2017). How FDA plans to help consumer capitalize on advances in science. https://www.blogs.fda.gov/fdavoice/index.php/tag/in-silico-tools/.

[10] Rubio-Viqueira B, Hidalgo M (2009). Direct *in vivo* xenograft tumor model for predicting chemotherapeutic drug response in cancer patients.

[11] Jung J (2014). Human tumor xenograft models for preclinical assessment of anticancer drug development.

[12] Wong H, Choo EF, Alicke B (2012). Antitumor activity of targeted and cytotoxic agents in murine subcutaneous tumor models correlates with clinical response.

[13] Sausville EA, Burger AM (2006). Contributions of human tumor xenografts to anticancer drug development.

[14] Becher OJ, Holland EC (2006). Genetically engineered models have advantages over xenografts for preclinical studies.

[15] Rocchetti M, Simeoni M, Pesenti E, De Nicolao G, Poggesi I (2007). Predicting the active doses in humans from animal studies: a novel approach in oncology.

[16] Bissery MC, Guenard D, Gueritte-Voegelein F, Lavelle F (1991). Experimental antitumor activity of taxotere (RP 56976, NSC 628503), a taxol analogue.

[17] Houghton PJ, Morton CL, Tucker C (2007). The pediatric preclinical testing program: description of models and early testing results.

[18] Corbett TH, White K, Polin L (2003). Discovery and preclinical antitumor efficacy evaluations of LY32262 and LY33169.

[19] Steiner P, Joynes C, Bassi R (2007). Tumor growth inhibition with cetuximab and chemotherapy in non-small cell lung cancer xenografts expressing wild-type and mutated epidermal growth factor receptor.

[20] Wong H, Vernillet L, Peterson A (2012). Bridging the gap between preclinical and clinical studies using pharmacokinetic–pharmacodynamic modeling: an analysis of GDC-0973, a MEK inhibitor.

[21] Hather G, Liu R, Bandi S (2014). Growth rate analysis and efficient experimental design for tumor xenograft studies.

[22] Lobo ED, Balthasar JP (2002). Pharmacodynamic modeling of chemotherapeutic effects: application of a transit compartment model to characterize methotrexate effects *in vitro*.

[23] Simeoni M, Magni P, Cammia C (2004). Predictive pharmacokinetic–pharmacodynamic modeling of tumor growth kinetics in xenograft models after administration of anticancer agents.

[24] Bueno L, De Alwis DP, Pitou C (2008). Semi-mechanistic modelling of the tumour growth inhibitory effects of LY2157299, a new type I receptor TGF-β kinase antagonist, in mice.

[25] Jumbe NL, Xin Y, Leipold DD (2010). Modeling the efficacy of trastuzumab-DM1, an antibody drug conjugate, in mice.

[26] Kogame A, Tagawa Y, Shibata S (2013). Pharmacokinetic and pharmacodynamic modeling of hedgehog inhibitor TAK-441 for the inhibition of Gli1 messenger RNA expression and antitumor efficacy in xenografted tumor model mice.

[27] Salphati L, Wong H, Belvin M (2010). Pharmacokinetic–pharmacodynamic modeling of tumor growth inhibition and biomarker modulation by the novel phosphatidylinositol 3-kinase inhibitor GDC-0941.

[28] Yamazaki S, Skaptason J, Romero D (2008). Pharmacokinetic–pharmacodynamic modeling of biomarker response and tumor growth inhibition to an orally available cMet kinase inhibitor in human tumor xenograft mouse models.

[29] Mould DR, Walz AC, Lave T, Gibbs JP, Frame B (2015). Developing exposure/response models for anticancer drug treatment: special considerations.

[30] Wong H, Belvin M, Herter S (2009). Pharmacodynamics of 2-[4-[(1E)-1-(hydroxyimino)-2,3-dihydro-1H-inden-5-yl]-3-(pyridine-4-yl)-1H-pyraz ol-1-yl]ethan-1-ol (GDC-0879), a potent and selective B-Raf kinase inhibitor: understanding relationships between systemic concentrations, phosphorylated mitogen-activated protein kinase kinase 1 inhibition, and efficacy.

[31] Lombardo F, Waters NJ, Argikar UA (2013). Comprehensive assessment of human pharmacokinetic prediction based on *in vivo* animal pharmacokinetic data, part 2: clearance.

[32] Lombardo F, Waters NJ, Argikar UA (2013). Comprehensive assessment of human pharmacokinetic prediction based on *in vivo* animal pharmacokinetic data, part 1: volume of distribution at steady state.

[33] Margolskee A, Darwich AS, Pepin X (2017). IMI – oral biopharmaceutics tools project – evaluation of bottom-up PBPK prediction success part 1: characterisation of the OrBiTo database of compounds.

[34] Li L, Gardner I, Dostalek M, Jamei M (2014). Simulation of monoclonal antibody pharmacokinetics in humans using a minimal physiologically based model.

[35] Lobo ED, Hansen RJ, Balthasar JP (2004). Antibody pharmacokinetics and pharmacodynamics.

[36] Wong H, Alicke B, West KA (2011). Pharmacokinetic–pharmacodynamic analysis of vismodegib in preclinical models of mutational and ligand-dependent Hedgehog pathway activation.

[37] Tate SC, Cai S, Ajamie RT (2014). Semi-mechanistic pharmacokinetic/pharmacodynamic modeling of the antitumor activity of LY2835219, a new cyclin-dependent kinase 4/6 inhibitor, in mice bearing human tumor xenografts.

[38] Palani S, Patel M, Huck J (2013). Preclinical pharmacokinetic/pharmacodynamic/efficacy relationships for alisertib, an investigational small-molecule inhibitor of Aurora A kinase.

[39] Betts AM, Haddish-Berhane N, Tolsma J (2016). Preclinical to clinical translation of antibody-drug conjugates using PK/PD modeling: a retrospective analysis of inotuzumab ozogamicin.

[40] Chen X, Haddish-Berhane N, Moore P (2016). Mechanistic projection of first in human dose for bispecific immuno-modulatory P-cadherin LP-DART – an integrated PK/PD modeling approach.

[41] Haddish-Berhane N, Shah DK, Ma D (2013). On translation of antibody–drug conjugates efficacy from mouse experimental tumors to the clinic: a PK/PD approach.

[42] Sapra P, Betts A, Boni J (2013). Preclinical and clinical pharmacokinetic/pharmacodynamic considerations for antibody–drug conjugates.

[43] Shah DK, Haddish-Berhane N, Betts A (2012). Bench to bedside translation of antibody–drug conjugates using a multiscale mechanistic PK/PD model: a case study with brentuximab-vedotin.

[44] Singh AP, Maass KF, Betts AM (2016). Evolution of antibody–drug conjugate tumor disposition model to predict preclinical tumor pharmacokinetics of Trastuzumab-Emtansine (T-DM1).

[45] Lindauer A, Valiathan CR, Mehta K (2017). Translational pharmacokinetic/pharmacodynamic modeling of tumor growth inhibition supports dose-range selection of the anti-PD-1 antibody pembrolizumab.

[46] Huck JJ, Zhang M, Mettetal J (2014). Translational exposure-efficacy modeling to optimize the dose and schedule of taxanes combined with the investigational Aurora A kinase inhibitor MLN8237 (alisertib).

[47] Terranova N, Germani M, Del Bene F, Magni P (2013). A predictive pharmacokinetic–pharmacodynamic model of tumor growth kinetics in xenograft mice after administration of anticancer agents given in combination.

[48] Choo EF, Ng CM, Berry L (2013). PK–PD modeling of combination efficacy effect from administration of the MEK inhibitor GDC-0973 and PI3K inhibitor GDC-0941 in A2058 xenografts.

[49] Mackey MC (2001). Cell kinetic status of haematopoietic stem cells.

[50] Pang WW, Price EA, Sahoo D (2011). Human bone marrow hematopoietic stem cells are increased in frequency and myeloid-biased with age.

[51] Craig M, Humphries AR, Mackey MC (2016). An upper bound for the half-removal time of neutrophils from circulation.

[52] Rankin SM (2010). The bone marrow: a site of neutrophil clearance.

[53] Friberg LE, Henningsson A, Maas H, Nguyen L, Karlsson MO (2002). Model of chemotherapy-induced myelosuppression with parameter consistency across drugs.

[54] Friberg LE, Sandstrom M, Karlsson MO (2010). Scaling the time-course of myelosuppression from rats to patients with a semi-physiological model..

[55] Zandvliet AS, Karlsson MO, Schellens JHM, Copalu W, Beijnen JH, Huitema ADR (2009). Two-stage model-based clinical trial design to optimize Phase I development of novel anticancer agents.

[56] Soto E, Keizer RJ, Trocóniz IF (2010). Predictive ability of a semi-mechanistic model for neutropenia in the development of novel anti-cancer agents: two case studies.

[57] Hansson EK, Friberg LE (2012). The shape of the myelosuppression time profile is related to the probability of developing neutropenic fever in patients with docetaxel-induced grade IV neutropenia.

[58] Pastor ML, Laffont CM, Gladieff L, Schmitt A, Chatelut E, Concordet D (2013). Model-based approach to describe G-CSF effects in carboplatin-treated cancer patients.

[59] Poikonen P, Saarto T, Lundin J, Joensuu H, Blomqvist C (1999). Leucocyte nadir as a marker for chemotherapy efficacy in node-positive breast cancer treated with adjuvant CMF.

[60] Stein A, Voigt W, Jordan K (2010). Chemotherapy-induced diarrhea: pathophysiology frequency and guideline-based management.

[61] Loriot Y, Perlemuter G, Malka D (2008). Drug insight: gastrointestinal and hepatic adverse effects of molecular-targeted agents in cancer therapy.

[62] Gibson RJ, Stringer AM (2009). Chemotherapy-induced diarrhoea.

[63] Benson AB, Ajani JA, Catalano RB (2004). Recommended guidelines for the treatment of cancer treatment-induced diarrhea.

[64] Gamucci T, Moscetti L, Mentuccia L (2014). Optimal tolerability and high efficacy of a modified schedule of lapatinib-capecitabine in advanced breast cancer patients.

[65] Potten CS, Loeffler M (1990). Stem cells: attributes, cycles, spirals, pitfalls and uncertainties. Lessons for and from the crypt.

[66] Bach SP, Renehan AG, Potten CS (2000). Stem cells: the intestinal stem cell as a paradigm.

[67] Nordgaard I, Mortensen PB (1995). Digestive processes in the human colon.

[68] Paulus U, Potten CS, Loeffler M (1992). A model of the control of cellular regeneration in the intestinal crypt after perturbation based solely on local stem cell regulation.

[69] Carulli AJ, Samuelson LC, Schnell S (2014). Unraveling intestinal stem cell behavior with models of crypt dynamics.

[70] Tomlinson IP, Bodmer WF (1995). Failure of programmed cell death and differentiation as causes of tumors: some simple mathematical models.

[71] Itzkovitz S, Blat IC, Jacks T, Clevers H, Van Oudenaarden A (2012). Optimality in the development of intestinal crypts.

[72] Van Leeuwen IM, Mirams GR, Walter A (2009). An integrative computational model for intestinal tissue renewal.

[73] Shankaran H, Cronin A, Barnes J (2017). Systems pharmacology model of gastrointestinal damage predicts species differences and optimizes clinical dosing schedules.

[74] Hecht JR (1998). Gastrointestinal toxicity or irinotecan.

[75] Gintant G, Sager PT, Stockbridge N (2016). Evolution of strategies to improve preclinical cardiac safety testing.

[76] Wolenski FS, Zhu AZ, Johnson M (2017). Fasiglifam (TAK-875) alters bile acid homeostasis in rats and dogs: a potential cause of drug induced liver injury.

[77] Sturla SJ, Boobis AR, Fitzgerald RE (2014). Systems toxicology: from basic research to risk assessment.

[78] Gao H, Korn JM, Ferretti S (2015). High-throughput screening using patient-derived tumor xenografts to predict clinical trial drug response.

[79] Dienstmann R, Vermeulen L, Guinney J, Kopetz S, Tejpar S, Tabernero J (2017). Consensus molecular subtypes and the evolution of precision medicine in colorectal cancer.

[80] Guinney J, Dienstmann R, Wang X (2015). The consensus molecular subtypes of colorectal cancer.

